# Ecological character displacement in the face of gene flow: Evidence from two species of nightingales

**DOI:** 10.1186/1471-2148-11-138

**Published:** 2011-05-24

**Authors:** Radka Reifová, Jiří Reif, Marcin Antczak, Michael W Nachman

**Affiliations:** 1 Department of Zoology, Faculty of Science, Charles University, Prague, Czech Republic; 2 Institute for Environmental Studies, Faculty of Science, Charles University, Prague, Czech Republic; 3 Department of Zoology and Laboratory of Ornithology, Faculty of Science, Palacký University, Olomouc, Czech Republic; 4 Department of Behavioural Ecology, Adam Mickiewicz University, Poznan, Poland; 5 Department of Ecology and Evolutionary Biology, University of Arizona, Tucson, USA

## Abstract

**Background:**

Ecological character displacement is a process of phenotypic differentiation of sympatric populations caused by interspecific competition. Such differentiation could facilitate speciation by enhancing reproductive isolation between incipient species, although empirical evidence for it at early stages of divergence when gene flow still occurs between the species is relatively scarce. Here we studied patterns of morphological variation in sympatric and allopatric populations of two hybridizing species of birds, the Common Nightingale (*Luscinia megarhynchos*) and the Thrush Nightingale (*L. luscinia*).

**Results:**

We conducted principal component (PC) analysis of morphological traits and found that nightingale species converged in overall body size (PC1) and diverged in relative bill size (PC3) in sympatry. Closer analysis of morphological variation along geographical gradients revealed that the convergence in body size can be attributed largely to increasing body size with increasing latitude, a phenomenon known as Bergmann's rule. In contrast, interspecific interactions contributed significantly to the observed divergence in relative bill size, even after controlling for the effects of geographical gradients. We suggest that the divergence in bill size most likely reflects segregation of feeding niches between the species in sympatry.

**Conclusions:**

Our results suggest that interspecific competition for food resources can drive species divergence even in the face of ongoing hybridization. Such divergence may enhance reproductive isolation between the species and thus contribute to speciation.

## Background

Understanding how interactions between closely related species affect their phenotypic evolution has long been of interest to evolutionary ecologists. It has been suggested that selection for reduced interspecific competition can lead to species divergence in areas of sympatry [1,2]. This process, known as ecological character displacement, has been suggested to be an important mechanism contributing to the origin of biological diversity [3-6]. In birds, the pattern of ecological character displacement is most often seen in body size and bill morphology [7-12]. Greater morphological divergence in sympatry can also be caused by natural selection against maladaptive interspecific hybridization, a process called reproductive character displacement (or reinforcement, when postzygotic isolation is incomplete) [1,13]. In birds, this primarily involves male signaling traits including bird song [14,15] and plumage [16]. On the other hand, under some conditions interspecific interactions can result in phenotypic convergence in sympatry [17-20]. The mechanisms leading to character convergence include competition for non-substitutable (essential) resources [21], selection to maintain interspecific territoriality [22] or heterospecific copying of acoustic or behavioral signals [19,23,24]. In addition, if reproductive isolation between the species is incomplete, introgressive hybridization may result in interspecific convergence in sympatry [23,25-28].

Phenotypic changes caused by interspecific interactions may play an important role in the process of speciation. Two kinds of outcomes are possible. First, character convergence of incipient species can increase the rate of interspecific hybridization, which could result in genetic fusion of both species [29]. Second, reproductive or ecological character displacement could facilitate the process of speciation by adding an additional degree of reproductive isolation between incipient species. The role of reproductive character displacement or reinforcement in increased isolation has been shown in many examples in hybridizing taxa [16,30-37]. Ecological character displacement can theoretically also lead to increased reproductive isolation in hybridizing taxa, and this can be both at the premating and at the postmating stage [5,38,39]. Nonetheless, examples of ecological character displacement in the course of speciation, when gene flow still occurs between the species, are relatively scarce [5,12,40].

The Thrush Nightingale (*Luscinia luscinia*, Linnaeus 1758) and the Common Nightingale (*Luscinia megarhynchos*, Brehm 1831) are suitable model species for studying phenotypic evolution during the process of speciation. These species diverged during Pleistocene climatic oscillations about 1.8 Mya [41]. The present range of *L. megarhynchos *extends from Southwestern Europe, across the Middle East to Central Asia. The distribution of *L. luscinia *is more northern and extends from Northeastern Europe to Northern Asia [42]. The ranges of both species overlap in a narrow hybrid zone running from north Germany, across Poland and Hungary to the Black sea [43]. The age of this hybrid zone is not known, but given its location within the northern part of Europe, we can assume that it arose after the last ice age, i.e. approximately 10,000 years ago or later [44]. Despite the overall morphological similarity, the species can be clearly distinguished by several wing feather characteristics and by plumage coloration [45]. In addition, *L. luscinia *is slightly larger relative to *L. megarhynchos *[46]. The species also show different song patterns, although *L. luscinia *males often sing like *L. megarhynchos *in the area of overlap [43,47]. Both species have similar ecological requirements. They occupy scrubby habitats and margins of broad-leaved forests with dense undergrowth, often near water [46]. However, some spatial segregation of territories has been observed in sympatry; *L. luscinia *tends to occur closer to water, while *L. megarhynchos *prefers drier habitats [43,48]. The species show strong assortative mating in sympatry. Nonetheless, mixed pairs occasionally arise and produce viable F_1 _hybrids [49,50]. Morphological studies suggest that approximately 5% of the birds in a sympatric population are F_1 _hybrids [49]. In accordance with Haldane's rule, F_1 _hybrid females are sterile, but F_1 _hybrid males are fertile [51] and can thus mediate gene flow between the species. Indeed, occurrence of interspecific gene flow has been documented between these species at multiple loci [41].

To investigate how ecological and reproductive interactions between the nightingale species affect their phenotypic evolution and the speciation process, we studied patterns of morphological variation in allopatric and sympatric populations of both species. Phenotypic variation in nightingales was first studied by Sorjonen [43]. He found that some morphological traits tended to diverge while others tended to converge in sympatry. However, these trends were not significant, and the study was based on limited sampling. Here, we expand on that work by carrying out extensive sampling across the whole sympatric region and adjacent parts of allopatric regions of both species. This sampling enables us to study whether the morphological changes between allopatric and sympatric populations result from interspecific interactions or from alternative processes such as gradual changes along large-scale geographical gradients [52,53]. Moreover, we performed DNA sequence analysis to control for the effect of recent interspecific hybridization on morphological variation.

## Methods

### Sampling

**Figure 1:**
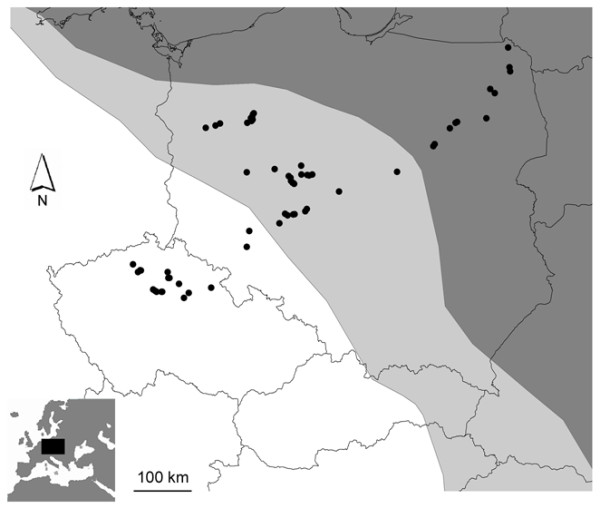
**Localities**. Map of localities where males of the Thrush Nightingale (*Luscinia luscinia*) and the Common Nightingale (*Luscinia megarhynchos*) were sampled. White - allopatric range of *L. megarhynchos*, dark grey - allopatric range of *L. luscinia*, light grey - range overlap of both species (i.e., sympatry). Species' ranges are redrawn with modifications from Sorjonen [43], Cramp [46] and Hagemeijer and Blair [54].

Nightingales were sampled in three regions: an allopatric region for *L. megarhynchos *(Czech Republic and south-western Poland), an allopatric region for *L. luscinia *(north-eastern Poland) and a sympatric region (central Poland) (Figure 1). The borders between sympatric and allopatric regions were determined following Sorjonen [43], Cramp [46], Hagemeijer & Blair [54] and adjusted after taking into account recent information from local ornithologists. The sampling was conducted in May 2007. All birds were males captured at the beginning of the breeding season (when territories are already established) in Ecotone mist nets with tape luring. The males were adults in the second calendar year or older (the age of each individual is provided in Additional file 1). In total, we trapped 173 males in 60 different localities (Figure 1; the exact geographic position of each locality is provided in Additional file 1). The species identity of sympatric individuals was determined according to species-specific morphological characteristics and confirmed by genetic analysis (see below). Three individuals were recognized as interspecific hybrids and were excluded from further analyses (see Results). The remaining 170 birds included 36 allopatric individuals of *L. megarhynchos*, 47 sympatric individuals of *L. megarhynchos*, 35 allopatric individuals of *L. luscinia *and 52 sympatric individuals of *L. luscinia*. The field work and manipulation with birds was approved by the Local Ethic Committee for Scientific Experiments on Animals in Poznan, Poland (permission no. 27/2008) and by the Ministry of Education, Youth and Sport of the Czech Republic (permission no.: 9833/2007-30).

### Morphological measurements

Each male was measured by JR using the same techniques [55] and equipment for all specimens. We measured several ecologically important traits including bill length (measured to skull), bill depth, bill width (both measured at frontal margin of nostrils), tarsus length (excluding heel joint), wing length, and body mass. All measurements are provided in Additional file 1.

### Genetic analysis

The species identity of sympatric individuals was determined from partial intronic sequences of two Z-linked genes, *ADAMTS6 *and *SPINZ-2*. Both loci were previously reported to harbor several species-specific single nucleotide polymorphisms (SNPs) that enable species identification [41]. We PCR amplified a 650 bp fragment of *ADAMTS6 *and a 950 bp fragment of *SPINZ-2*. These fragments were sequenced in both directions and base composition has been determined at positions carrying previously identified species-specific SNPs. Primer sequences as well as PCR conditions are described in Storchová *et al. *[41]. Although we do not know the exact chromosomal position of these genes in nightingales, both genes are separated from each other by more than 20 Mb both in chicken and zebra finch, suggesting that they may also be this far apart in nightingales. The obtained sequences have been deposited in GenBank under accession numbers [GenBank: GQ388014-GQ388115, HM146205-HM146304].

### Statistical analyses

We used a principal component (PC) analysis on the correlation matrix of the six morphological measurements, to reveal new variables (PC1-PC6) that are independent of each other. Patterns of variation in these principal components were then investigated using two-way factorial analysis of variance (ANOVA), where species (i.e., *L. luscinia *or *L. megarhynchos*) and region (i.e., allopatry or sympatry) were treated as factors. A significant interaction between the factors identifies species-specific phenotypic changes from allopatry to sympatry, which may reflect a pattern of convergent or divergent character displacement in sympatry [56,57].

Significant interactions between the effect of species and region can also reflect change of a trait along a geographical gradient [52,53]. To investigate the role of possible geographical gradients on morphological changes, we performed analysis of covariance (ANCOVA), where geographical coordinates (i.e., latitude and longitude) of the trapping sites were included as covariates. The significant interaction between the effect of species and region in the model with geographical coordinates implies that ecological interactions between the species contribute to the morphological changes between allopatry and sympatry. A test of the significance of the magnitude of the shift between sympatry and allopatry within species was performed using *a priori *contrasts in the models with significant interaction.

## Results

### Genetic analysis of sympatric individuals

We sequenced 102 sympatric males and observed three individuals of hybrid ancestry. Two of these individuals had heterozygous genotypes for both of the genes analyzed (i.e., one haplotype was of *L. megarhynchos *origin, while the other of *L. luscinia *origin). These individuals also showed intermediate phenotypes; in some traits they resembled *L. megarhynchos*, while in others they resembled *L. luscinia*. This suggests that they are F_1 _hybrids or early backcross individuals. The third hybrid individual was heterozygous at *SPINZ-2 *and was homozygous for *L. luscinia *alleles at *ADAMTS6*. This individual was morphologically indistinguishable from *L. luscinia *and represents a backcross or later-generation hybrid. These results suggest that about 3% of the nightingales in sympatry are of mixed ancestry. This is likely to be a minimum estimate since some backcross or later-generation progeny may not be detected using only two loci on the Z chromosome. To minimize the effect of recent hybridization on morphological variability, these three hybrids were excluded from the morphological analyses below.

### Patterns of morphological variation in allopatric and sympatric populations of nightingales

Many morphological traits are correlated with each other and a change in one trait can thus affect other traits. To overcome this problem, we used PC analysis to transform the original morphological measurements into six principal component axes (PC1-PC6) that are not correlated with each other. Explained variation, eigenvalues and factor loadings for each PC axis are shown in Table 1. The first three axes can be interpreted as follows: PC1 largely reflects variability in overall body size (note that PC1 shows positive correlation with all body size measurements including body mass and wing length), PC2 mainly reveals variability in bill and tarsus length, and PC3 corresponds to variability in relative bill size when compared to body size (note that PC3 positively correlates with all bill size measurements, although the strongest is correlation with bill width, and negatively correlates with all body size measurements).

**Table 1:** Explained variation, eigenvalues and factor loadings for six principal component axes (PC1-PC6)

	PC1	PC2	PC3	PC4	PC5	PC6
Explained variation (%)	34.8	19.6	16.6	12.6	10.9	05.5
Eigenvalues	2.09	1.17	1.00	0.76	0.66	0.33
						
Bill length	0.28	0.74	0.17	0.55	-0.19	0.06
Bill width	0.43	-0.12	0.76	-0.29	-0.36	0.02
Bill depth	0.67	0.07	0.32	0.02	0.67	-0.04
Tarsus length	0.33	0.66	-0.32	-0.59	-0.02	0.07
Wing length	0.77	-0.38	-0.29	0.12	-0.09	0.40
Body mass	0.82	-0.16	-0.32	0.08	-0.20	-0.40

**Figure 2:**
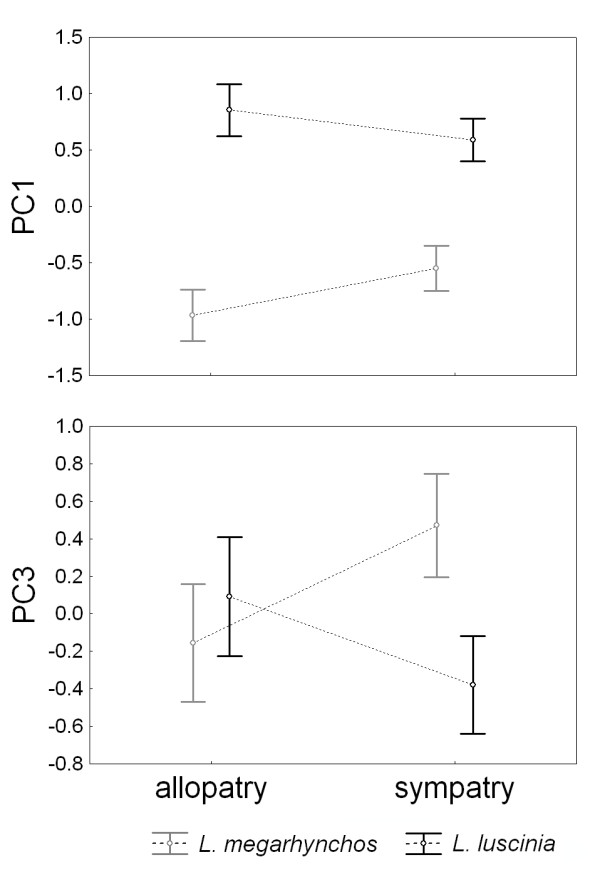
**Patterns of variation in PC1 and PC3 in allopatric and sympatric populations of nightingales**. Nightingales have converged in PC1 and diverged in PC3 in sympatry. Means ± 95% confidence intervals are shown.

To assess the effect of species (i.e., *L. luscinia *or *L. megarhynchos*), region (i.e., allopatry or sympatry) and their interactions on the morphological variability in nightingales, we performed two-way factorial ANOVA separately for the five PC axes explaining more than 10% of the variability in the morphological data (Table 2). The analyses revealed strong effects of species in PC1 (F = 190.05, p < 0.001) and PC2 (F = 31.26, p < 0.001), and weaker but still significant effect of species in PC3 (F = 4.11, p = 0.044) (Table 2). This is consistent with previous descriptions of diagnostic traits for these species [40] showing that *L. luscinia *is generally larger compared to *L. megarhynchos *(this is reflected in PC1), and that *L. megarhynchos*, although smaller, has a relatively long tarsus and bill (this is reflected in PC2). Importantly, we found significant interactions between the effect of species and region in PC1 (F = 10.14, p = 0.002) and PC3 (F = 13.67, p < 0.001) (Table 2). The interactions remained significant even after applying a Bonferroni correction for multiple testing which adjusts significant p-value to 0.01. The revealed interaction reflects character convergence in sympatry in the case of PC1 and character divergence in sympatry in the case of PC3 (Figure 2). Our results thus suggest that the nightingales have converged in overall body size and diverged in relative bill size in sympatry.

**Table 2:** Influence of species, region and their interactions on changes in particular principal components

	species		region		species × region	
	F	p	F	p	F	p
PC1	190.05	**<0.001**	0.49	0.484	10.14	**0.002**
PC2	31.26	**<0.001**	0.55	0.458	3.28	0.072
PC3	4.11	**0.044**	0.28	0.697	13.67	**<0.001**
PC4	0.42	0.516	0.48	0.489	0.98	0.324
PC5	1.02	0.313	0.94	0.333	1.17	0.281

Only principal components explaining more than 10% of variability in morphological data were included in the analysis. Degrees of freedom were 1 for each factor and 166 for error in all analyses. Significant p-values (p < 0.05) are indicated in bold.

### Effect of environmental gradients on morphological variation

**Figure 3:**
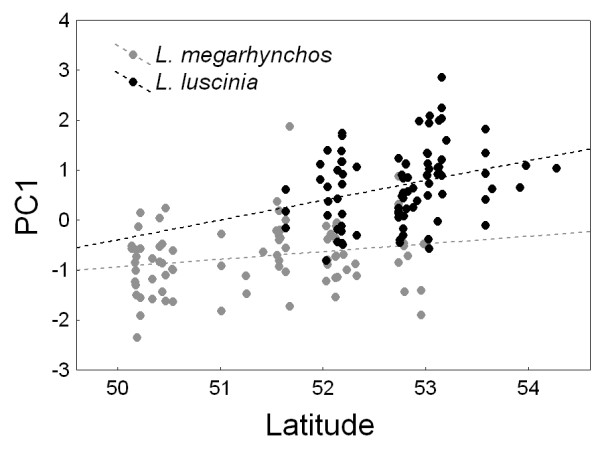
**Relation between PC1 and latitude**. PC1 increases with increasing latitude in both species of nightingales.

Patterns of character divergence or convergence in sympatry can arise not only due to interspecific interactions, but also due to changes along an environmental gradient [52,53]. To investigate the effects of geographic gradients on the observed patterns of morphological variability in PC1 and PC3, we performed ANCOVA with geographic coordinates (latitude and longitude) included as covariates in the models. We found a strong effect of latitude (F = 128.57, p < 0.001) and a weaker but still significant effect of longitude (F = 6.64, p = 0.011) on the variability in PC1 (Table 3). Figure 3 shows that PC1 increases with increasing latitude in both species. The interaction between species and region was no longer significant for PC1 (F = 0.30, p = 0.585) when these geographical variables were included into the model (Table 3). This suggests that the observed convergence of PC1 in sympatry reflects changes along a geographical gradient rather than between-species interactions. Longitude also had a significant effect on the variability in PC3 (F = 9.10, p = 0.003). However, the interaction between the effect of species and region remained significant (F = 5.53, p = 0.020) even when latitude and longitude were included as variables into the model (Table 3). This suggests that interspecific interactions contribute significantly to the observed character divergence for PC3 in sympatry.

**Table 3:** Influence of geographical gradients, species, region and their interactions on changes in PC1 and PC3

	latitude		longitude		species		region		species × region	
	F	p	F	p	F	p	F	p	F	p
PC1	128.57	**<0.001**	6.64	**0.011**	55.44	**<0.001**	0.14	0.713	0.30	0.585
PC3	0.83	0.364	9.10	**0.003**	12.52	**<0.001**	6.55	**0.011**	5.53	**0.020**

Degrees of freedom were 1 for each factor and 164 for error in all analyses. Significant p-values (p < 0.05) are indicated in bold.

**Figure 4:**
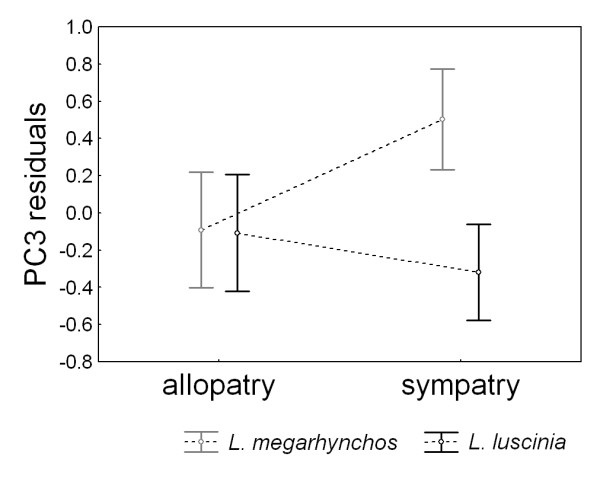
**Variation of PC3 residuals in allopatric and sympatric populations of nightingales**. The graph shows variation of PC3 residuals where the effects of latitude and longitude are controlled for. The Common Nightingale (*Luscinia megarhynchos*) shows greater change in PC3 residuals between sympatry and allopatry than the Thrush Nightingale (*L. luscinia*). Means ± 95% confidence intervals are shown.

To explore whether the divergence of PC3 in sympatry was caused by a morphological shift in both species or only in one species, we performed an analysis of contrasts using the model in which geographical variables were included. This analysis revealed a significant difference in PC3 from allopatry to sympatry in *L. megarhynchos *(t = -4.14, p < 0.001) but not in *L. luscinia *(t = 1.03, p = 0.306). The difference in PC3 between the regions in *L. megarhynchos *is caused by an increase in PC3 in sympatric population of this species as can be seen in the pattern of changes of PC3 residuals (i.e., after removing the effects of latitude and longitude) between the regions (Figure 4).

### Do changes in bill morphology result from sorting of pre-existing variation or from *in situ *evolution of a novel phenotype?

**Figure 5:**
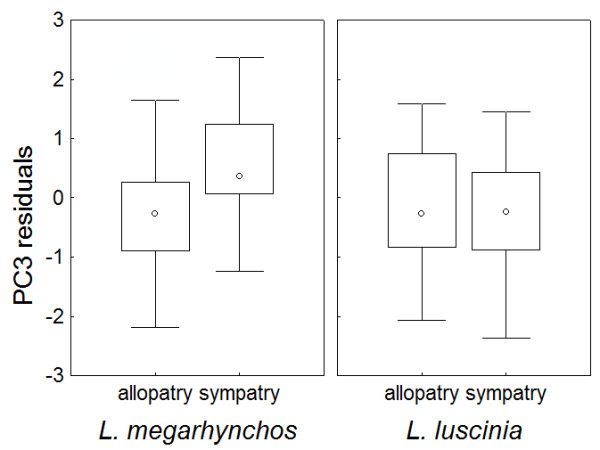
**Distributions of PC3 residuals in allopatric and sympatric populations of nightingales**. The graph shows distributions of PC3 residuals where the effects of latitude and longitude are controlled for. Boxes with the middle point represent median and 25% - 75% quartiles. The whiskers show non-outlier ranges.

We were further interested in whether the observed increase in PC3 in sympatric populations of *L. megarhynchos *results from sorting of pre-existing variation or *in situ *evolution of a novel phenotype [58]. In the first case, the divergent trait is present in an ancestral allopatric population, but its frequency increases after secondary contact in sympatry as a result of competitively mediated selection or biased colonization and extinction, which might not represent ecological character displacement [40]. In the second case, the divergent trait appears as a new trait in sympatry in response to the presence of heterospecific competitors. To distinguish between these two scenarios, we compared the distributions of PC3 residuals in sympatric and allopatric populations of both species (Figure 5). The hypothesis of sorting of pre-existing variation predicts that the range of PC3 values in allopatric populations (which represent the source for colonization of sympatric populations) will be larger and will encompass the range of PC3 values in sympatry for the same species. In contrast, the evolutionary shift hypothesis predicts different ranges of PC3 values between allopatry and sympatry. Our data show that the non-outlier range of PC3 values for sympatric population of *L. megarhynchos *is shifted, and maximum values reach well beyond the non-outlier range observed for allopatric population of the same species (Figure 5). The finding that PC3 values in sympatry are not a subset of PC3 values in allopatry for *L. megarhynchos *suggests that *in situ *evolution of a novel phenotype has occurred in this species.

## Discussion

To study how interactions between two closely related species of nightingales affect their phenotypic evolution, we analyzed patterns of morphological variation in allopatric and sympatric populations of both species. Our analysis revealed two main patterns of morphological change. First, nightingales have converged in overall body size (as reflected in PC1) in sympatry. Second, nightingales diverged in relative bill size when compared to body size (as reflected in PC3) in sympatry. This divergence was asymmetric and was caused mainly by increased bill size in *L. megarhynchos*. Closer analysis of morphological variation along geographical gradients revealed that the convergence in overall body size was mainly caused by increasing body size with increasing latitude (Figure 3), a phenomenon known as Bergmann's rule [59-61]. Interspecific interactions did not have a significant effect on the convergence in body size (Table 3). On the other hand, interspecific interactions contributed significantly to the divergence in relative bill size even after controlling for the effects of geographical gradients (Table 3). Below, we argue that the observed divergence in relative bill size is most likely caused by interspecific competition for food resources and discuss how this ecological character displacement might facilitate speciation in nightingales.

### Evidence for ecological character displacement in nightingales

Schluter & McPhail [40] summarized six criteria for demonstrating the occurrence of ecological character displacement. (1) The pattern should not occur by chance. (2) Sites of sympatry and allopatry should not differ greatly in food, climate, or other environmental features affecting the phenotype. (3) Morphological differences should reflect differences in resource use. (4) There must be independent evidence for competition. (5) Enhanced differences should result from actual evolutionary shifts, not from the biased colonization and extinction of similar-sized individuals. (6) Phenotypic differences should have a genetic basis. Meeting all of these criteria is usually quite difficult, and there are surprisingly few studies where alternative explanations for sympatric divergence have been ruled out and interspecific competition for food resources has been proven as a causal mechanism [12,40,62,63]. In this study, we assemble evidence that at least partially satisfies four of these criteria (2-5).

Character displacement is typically demonstrated as a greater between-species morphological difference in sympatry than in allopatry. Such a pattern can, however, also arise if sites of sympatry and allopatry differ in environmental features affecting the phenotype (criterion 2). This is often caused by environmental gradients across species ranges. Our study area is located within central Europe, for which southwest-to-northeast climatic gradients are characteristic [64]. We sought to disentangle the effects of environmental gradients and interspecific interactions by incorporating geographical variables (latitude and longitude) in statistical models. This approach removes the effects of geographical gradients in all directions. We found that the interspecific interactions contribute significantly to the enhanced differences in bill morphology in sympatry even when the effects of environmental gradients were controlled for. It is thus unlikely that the observed changes in bill morphology are caused by different environmental features or by different food supplies in sympatric and allopatric regions.

If phenotypic differences in sympatric populations are caused by ecological character displacement, they should reflect differences in resource use (criterion 3). Bill morphology is generally closely linked to resource use in birds [65] and determines the type and size of the food as well as feeding strategies [11,12]. This is very likely also true for nightingales. Although both species of nightingales have a similar diet in general - they feed on small invertebrates on the ground in dense shrubby vegetation [46] - it is possible that minor differences in diet have evolved between the species in sympatry. These differences could be caused by either a separation of their feeding niches in sites where both species co-occur or by different food supplies in different microhabitats [66]. Territories of *L. luscinia *tend to occur in wetter sites in the region of sympatry, while *L. megarhynchos *is more frequent in drier places, probably due to displacement by interference competition [43,48]. In addition, Stadie [51] observed slightly different feeding strategies of the two species in sympatry. Whereas *L. megarhynchos *fed almost exclusively on the ground, *L. luscinia *was able to catch flying insects and was observed more frequently foliage gleaning [51]. These observations suggest that both niche separation within the same habitat and habitat segregation occur in sympatric populations of nightingales and might contribute to bill size divergence in sympatry.

Bill morphology in passerines could also be affected by song characteristics, such as frequency, harmonic content and temporal patterning [67-70]. Thus, an alternative explanation for bill size differentiation in nightingales would be a change of song in sympatry. Such change could be driven for example by selection against maladaptive hybridization, a phenomenon known as reproductive character displacement or reinforcement. This has been documented in African Tinker birds [15]. However, such an explanation is unlikely in this study system since song convergence rather then divergence occurs in sympatric populations of nightingales [43,47,71]. Moreover, song convergence in nightingales is caused by song change in *L. luscinia*, but not in *L. megarhynchos*, in contrast to the pattern that we observed in bill morphology.

Independent evidence for competition between species needs to be demonstrated to make a compelling case for ecological character displacement (criterion 4). Both species of nightingales have very similar habitat requirements [46] and show interspecific territoriality in sympatry [43]. Moreover, playback experiments have demonstrated that males of both species respond aggressively to heterospecific songs in sympatry [43]. This suggests that interspecific competition for resources is present in the two nightingale species. The role of interspecific competition in bill size divergence in nightingales is also supported by the observed asymmetry in morphological change in our data. Asymmetrical character displacement is expected if one species suffers higher costs during interspecific interactions; this species should diverge more than the other species [72]. In this study system, *L. megarhynchos *is the weaker competitor as is suggested by four observations. First, *L. megarhynchos *has a smaller body size compared to *L. luscinia *[46]. Second, *L. megarhynchos *shows partial habitat shift in sympatry [43]. Third, *L. megarhynchos *responds aggressively to the heterospecific song less often than *L. luscinia *[43]. Fourth, the zone of sympatry is slowly moving in the south-west direction towards the area of *L. megarhynchos*, which could be the result of dominance of *L. luscinia *in interspecific competition [73]. In accordance with the lower competitiveness of *L. megarhynchos*, this species shows significant shift in bill size between sympatry and allopatry, while the bill size of *L. luscinia *does not differ between the regions. This result is consistent with the idea that interspecific competition drives the bill size differentiation in nightingales.

Finally, character displacement should result from a true evolutionary shift, not from biased colonization and extinction of similarly sized individuals (criterion 5). For example, it is possible that sympatric regions were colonized preferentially by *L. megarhynchos *with large bills or that *L. megarhynchos *with small bills went extinct in sympatry due to reasons other than competitively mediated selection. We addressed this question by comparing the distributions of bill size values (as reflected in PC3 residuals after removing the effects of latitude and longitude) in sympatric and allopatric populations within the same species. We found that the non-outlier range of bill size values for sympatric population of *L. megarhynchos *reaches beyond the non-outlier range observed for allopatric population of the same species (Figure 5). This suggests that the observed increase in bill size in sympatric population of *L. megarhynchos *is caused by *in situ *evolution of a novel phenotype and thus represents a real evolutionary shift rather than biased colonization and extinction of individuals with certain phenotypes.

These observations suggest that ecological character displacement is likely to be the causal mechanism underlying morphological differences between sympatric populations of the two nightingale species. Nonetheless, several issues still need to be addressed to provide more direct evidence for ecological character displacement. First, the observed divergence in bill morphology should be demonstrated on additional independent populations to rule out the possibility that the pattern is caused by chance (criterion 1). Second, a direct link between bill size and food preferences should be established (criterion 3). Third, the relationship between bill size and the level of interspecific competition in sympatric populations should be demonstrated (criterion 4). Finally, it remains to be shown that the divergence of sympatric populations is genetically based (criterion 6), although some non-genetic changes may also reflect ecological character displacement as discussed below.

### Ecological character displacement in the face of gene flow: result of natural selection or phenotypic plasticity?

Ecological character displacement has often been regarded as a post-speciation event that occurs after the completion of reproductive isolation between incipient species [1-3,13,38]. In species where hybridization is common, interspecific gene flow can hinder ecological differentiation. In this study, we found that at least 3% of sympatric nightingales represent hybrids. In addition, gene flow between the species has been documented at multiple loci [41]. This raises the question of how morphological divergence in sympatric populations of nightingales is maintained and why it is not erased by interspecific gene flow.

One possible explanation is simply that natural selection has a stronger effect on allele frequencies at loci that are responsible for bill size variability than does the rate of interspecific gene flow. This can be thought of in the context of models of migration-selection balance. Alleles at loci controlling bill size will be introduced due to gene flow from the sister species, and will be removed due to selection. Under a number of simplifying assumptions, the equilibrium frequency (*q*) for a dominant deleterious allele introduced by migration at rate *m *and removed by selection of magnitude *s *is given by *q = m/s *[74]. In nightingales, most hybridization may not lead to gene flow since F_1 _females are sterile [51]. In our study, only one bird was a later-generation hybrid. If we take this as a very rough upper estimate of the level of gene flow (m = 0.01), then a 10% selective cost (s = 0.1) would be sufficient to keep introduced alleles at a relative low frequency (i.e., 10%). This very rough calculation is only meant to illustrate that the degree of gene flow is sufficiently low that strong selection could still maintain different allele frequencies for traits of ecological importance.

In fact, previous work suggested that this sort of selection regularly acts against alleles introduced by migration between these species [41]. That study showed that introgression between the nightingale species is significantly lower on the Z chromosome than on the autosomes, suggesting that selection acts against mis-matched Z-linked loci [41]. Indirect estimates of the overall migration rate from patterns of DNA sequence variation analyzed under an isolation-with-migration model [75] were on the order of 10^-7 ^[41], many orders of magnitude lower than the proportion of later-generation hybrids (10^-2^) observed in this study. This large difference suggests that many hybrid individuals may not contribute substantially to gene flow, perhaps because they have lower fitness. If so, then even weak selection might be sufficient to drive the evolution of bill shape differences in sympatry.

Why do alleles that increase bill size in sympatric *L. megarhynchos *not spread into allopatric populations? One possible explanation is that a larger bill is less optimal then the ancestral pre-displacement phenotype in allopatry. Indeed, character displacement might represent a "best-of-a-bad-situation", sensu [76], in that it lessens interspecific competition, but at a cost of reduction in other fitness parameters [77]. Such fitness trade-offs can generate a selective barrier to gene flow between sympatric and allopatric populations because individuals from either population will be disadvantaged in the alternate population [78].

Phenotypic plasticity could provide an alternative explanation for the maintenance of phenotypic differences in the presence of gene flow. Phenotypic plasticity is the ability of an organism to change its phenotype in response to environmental stimuli [79,80]. Since this is a non-genetic response, interspecific gene flow should not affect morphological differences caused by phenotypic plasticity. Recent evidence suggests that phenotypic plasticity in bill morphology can occur in response to poor conditions during development. Gil *et al. *[81] have demonstrated that nestlings of the Spotless Starling (*Sturnus unicolor*) develop larger bills and smaller body size in poor feeding conditions. Bill size of nestlings (especially the gape width) is an important determinant of food distribution among nestlings [82]. Gil *et al. *[81] thus suggested that growth of the bill could be favored over growth of overall body size when feeding conditions worsened. In nightingales, *L. megarhynchos*, which is the weaker competitor, might be forced to low-quality territories with reduced food availability in sites where both species co-occur. Nutritional stress could then cause increased bill size in nestlings and, at maturity, of the adult birds.

Morphological differences caused by phenotypic plasticity are traditionally not considered character displacement, since they do not have a genetic basis (although the tendency to express different phenotypes in varying environmental conditions can be determined genetically) and thus cannot be inherited (criterion 6). Interesting exceptions represent cases where plastic phenotypic change is transmitted to the next generation through maternal effect [83,84]. Recent studies, however, indicate that if plastic phenotypic change is adaptive (for example if it leads to change in food choice, which in turn reduces interspecific competition), it can eventually be stabilized by the evolution of genetic differences through a process known as genetic assimilation [85]. Phenotypic plasticity could thus facilitate the evolution of character displacement in the presence of gene flow [80]. Further developmental studies of nightingales raised on low and high quality food should provide more insight into the proximate mechanisms responsible for the divergence in bill size in these species.

## Conclusions

Darwin [86] was the first who highlighted the importance of competition in speciation, though his idea was not widely accepted. The major objection was that divergence through competition cannot occur without prior evolution of reproductive isolation between incipient species, because gene flow would preclude any divergence [38,87]. Only recently, theoretical studies have indicated that competitively mediated divergence could be possible even in the face of gene flow [88-90]. Yet, empirical examples of such process are still scarce and involve mainly species where hybridization is relatively rare [5,12,40,91]. Our results add to this evidence and suggest that selection for reduced competition could drive species divergence if gene flow is relatively low (specifically, if gene flow, *m*, is lower than selective cost, *s*, see above). In such situations, ecological character displacement can facilitate speciation by enhancing reproductive isolation. This might happen in several different ways. First, hybrids with intermediate phenotype might be exposed to higher levels of interspecific competition and thus be forced to live in marginal niches, increasing the degree of extrinsic postzygotic isolation. Second, separation of ecological niches may lead to reduced contacts between the species and thus increase the degree of prezygotic isolation. Third, if ecological divergence has a genetic basis, it should be associated with divergence in genomic regions underlying ecologically important traits, such as bill shape in nightingales. Such genetic differentiation could be accompanied by accumulation of genetic incompatibilities leading to reduced fertility or viability of hybrids and thus increase the degree of intrinsic postzygotic isolation. Further studies combining genetic, morphological and ecological data will help elucidate the role of bill size divergence in reproductive isolation between the nightingale species.

## Authors' contributions

RR contributed to data collection in the field, carried out molecular analyses and drafted the manuscript, JR collected the data in the field, performed the statistical analyses and was involved in drafting the manuscript, MA contributed significantly to data collection in the field, MWN was involved in drafting the manuscript. All authors read and approved the final manuscript.

## Supplementary Material

Additional file 1 **Geographic position and morphological measurements of individual samples**. List of all analyzed nightingale individuals including information about their geographic position and morphological measurements. Click here for file
